# Statutory regulation in somatology: a public health, professional, and decolonial imperative in South Africa

**DOI:** 10.3389/fpubh.2026.1765187

**Published:** 2026-02-12

**Authors:** Nomakhosi Mpofana, Michael Paulse, Dorinda Borg, Sphiwe Mbongeni Tshabalala, Judith Fungisani, Mokgadi Ursula Makgobole, Mandy Thomas

**Affiliations:** 1 Department of Somatology, Durban University of Technology, Durban, South Africa; 2 Graduate School of Business, The University of Cape Town, Cape Town, South Africa; 3 Central University of Technology, Bloemfontein, South Africa; 4 Department of Wellness Sciences, Cape Peninsula University of Technology, Cape Town, South Africa

**Keywords:** clinical risk, decoloniality, professional demarcation, public health, somatology, South Africa, statutory regulation

## 1 Introduction

### 1.1 A Profession at a Precipice

Somatology in South Africa occupies a paradoxical position. Evolving from vocational beauty therapy into a rigorous university discipline, it now encompasses scientifically grounded curricula in anatomy, dermatology, and holistic health management, accredited at National Qualifications Framework (NQF) Levels 6 through 8. This academic rigor equips somatologists to perform complex, minimally invasive procedures, from laser therapies to chemical peels, that carry inherent risks of permanent physical harm. Despite this clinical profile, the profession remains unregulated by statute. This legislative omission creates a dangerous environment where the legal distinction between a university-trained somatologist and a short-course certificate holder is non-existent.

The consequences are severe. Global data indicate that aesthetic procedures performed by underprepared practitioners significantly increase adverse events, including vascular occlusion and burns (1). In South Africa, this risk is amplified; without specialized competency in treating Skin of Color, lasers and chemical peels disproportionately result in permanent post-inflammatory hyperpigmentation and scarring (2, 3). Beyond physical harm, this lack of governance facilitates the devaluation of advanced qualifications and reinforces systemic inequities. Statutory regulation is the only viable mechanism to safeguard public health and stabilize professional identity.

## 2 The evolution and demarcation of somatology

To understand the necessity of regulation, one must contextualize Somatology as a clinical discipline that has outpaced its governance. The profession's foundation was established under the Technikon system, standardized by the Certification Council for Technikon Education (SERTEC) (4). A paradigm shift occurred in the mid-1990s when the discipline rebranded to “Somatology” to explicitly differentiate university-qualified practitioners from vocational “beauty therapists” (5).

This nomenclature change signaled a transition toward a holistic, scientifically grounded pedagogy (6). Governance was further solidified in 2008 via the National Qualifications Framework Act No. 67, placing qualifications under the South African Qualifications Authority (SAQA). Consequently, the structure matured into a tiered system: Diploma (NQF 6), Advanced Diploma (NQF 7), and Postgraduate Diploma (NQF 8) (7).

A central contradiction now exists: while the education sector has successfully professionalized Somatology into a high-level academic field, the professional regulatory framework remains non-existent. We have a cadre of NQF Level 8 professionals who remain legally indistinguishable from unregulated operators, exposing a disconnect between the state's investment in education and its failure to protect the profession.

## 3 The tripartite imperative for statutory intervention

### 3.1 Public health and safety: mitigating clinical risk

The foremost imperative of regulation is mitigating preventable harm. Effective governance requires a tiered approach, distinguishing low-risk aesthetics from high-risk clinical modalities. Currently, any individual, regardless of competency, can legally offer invasive procedures such as medical needling or laser ablation.

In South Africa, where a significant demographic presents with Fitzpatrick Skin Types IV–VI, the unsupervised application of high-energy devices frequently results in permanent injury. These injuries often remain unreported due to the lack of a formal complaints' mechanism; voluntary associations lack the authority to revoke practicing rights.

International frameworks offer a roadmap. Models like the UK's Joint Council for Cosmetic Practitioners (JCCP) and Australia's radiation licensing demonstrate that restricting high-risk procedures to credentialed professionals reduces adverse events (8). Local research confirms the appetite for such protection: 75% of somatology practitioners and 89% of medical professionals agree that statutory registration is essential (9). A statutory council with powers of inspection and discipline is the only vehicle capable of creating a mandatory standard of care.

### 3.2 Professional integrity and economic justice

The regulatory vacuum systematically undermines the Somatology qualification and enables labor exploitation. Graduates with years of clinical study often face wage parity with vocational certificate holders. According to Mpofana's personal communication in the Sunday Times, November 2025, this financial disconnect deters talent retention (10). Moreover, the industry retains an exploitative architecture where capital ownership is concentrated, while the workforce, predominantly Black women, remains confined to low-wage roles (11). The absence of a statutory or professional regulatory body presents a significant challenge in anchoring workforce demographics to an official statistical labor report, as no centralized records of registered Somatology practitioners currently exist. In response to this structural data gap, demographic patterns are inferred from national higher education participation data, specifically the Council on Higher Education (CHE, 2025) (12) report on first-time entering students, enrolments, and completions across public higher education institutions. Given that Universities of Technology constitute the primary training pipeline for Somatology qualifications, these data provide a credible and transparent proxy for understanding the profession's demographic composition.

Statutory regulation provides the mechanism to disrupt this cycle. By enforcing “Protection of Title” and linking high-value clinical scopes of practice to NQF levels, a council would legally mandate the differentiation between a Somatologist and a vocational operator. This legal formalism is a prerequisite for standardized remuneration guidelines and professional autonomy. Furthermore, a lack of regulation creates barriers to entry. While 66% of Somatologists receive referrals from complementary practitioners, only 39% of medical professionals refer patients to them, likely due to liability concerns (9). By establishing Somatology as a registered healthcare profession, a Council can advocate for sector-specific funding codes and allow practitioners to claim from medical aids. This shifts the economic model from precarious entrepreneurship to sustainable practice, moving regulation beyond technical oversight to a tool for economic justice.

### 3.3 Decolonial transformation

Decoloniality is defined here as the active dismantling of Eurocentric structures governing the profession. The South African beauty industry arguably remains a site where historical hierarchies persist, often cantering Eurocentric standards (13).

This bias is operationalized in clinical ways. Curricula often rely on white imagery for pathology demonstration, creating a pedagogical gap in diagnosing conditions on darker skin tones. Furthermore, core technologies like lasers were originally calibrated for Fitzpatrick skin types I–III (14). The resulting exclusion of darker-skinned clients or their injury is a failure of the self-regulatory model.

Statutory regulation provides a mechanism to address this by enforcing new accreditation criteria. A council would have the authority to mandate that accredited curricula include specific modules on dermatological conditions in Skin of Color and require that high-risk devices be FDA/CE cleared specifically for Fitzpatrick skin types IV–VI before local approval.

## 4 Addressing counterarguments and the informal sector

Skepticism regarding regulation often stems from concerns about bureaucratic inefficiency and the stifling of enterprise. These apprehensions must be addressed through intentional design. Regulation establishes a safety framework that preserves autonomy (15). To mitigate administrative burdens, the Council should implement digital-first registration and a risk-based tiered licensing model, concentrating oversight on invasive modalities (16).

Crucially, this transition must not disenfranchise small business owners or informal practitioners. To prevent regulation from becoming exclusionary, the framework must include a robust Recognition of Prior Learning (RPL) mechanism. This allows experienced practitioners to demonstrate competency through practical assessment rather than purely academic certification. Additionally, a “grandfathering” clause for established businesses could provide a grace period, ensuring regulation uplifts the existing workforce rather than criminalizing livelihoods.

Governance must be inclusive. The council's composition must guarantee representation for practitioners, educators, and consumer advocates from historically marginalized groups. This is a substantive requirement for decolonial transformation, ensuring the council acts to dismantle systemic inequality rather than reproduce it.

## 5 Limitations and future research

This study is limited by the absence of a formal statutory or regulatory body for the Somatology profession in South Africa, which, in turn, leads to a lack of a centralized repository for workforce data, employment conditions, or practitioner demographics. As a result, traditional labor-market statistics are limited. To maintain academic rigor, the study deliberately omits anecdotal or verbally reported practitioner experiences, which can be biased and are not independently verifiable. Instead, the analysis depends on national VitalStats: Public and Private Higher Education CHE, 2025 (12), and publicly available gray literature as reliable proxy indicators (11).

Future research will fill this gap through an empirical study involving structured interviews with Somatology practitioners, educators, employers, and relevant stakeholders. This work will collect primary data on professional practices, labor conditions, and workforce composition, allowing for more comprehensive national analysis and meaningful comparisons with international regulatory frameworks. Such empirical evidence will be essential in guiding policy development, statutory registration, and fair professional governance within South Africa's Somatology sector.

## 6 Conclusion

The evidence unequivocally identifies the regulatory vacuum in South African Somatology as a critical policy failure, one that endangers public health while entrenching post-apartheid inequality. Consequently, the authors advocate for the immediate opening of a register under the AHPCSA. To operationalize this, the Department of Health must urgently initiate a Regulatory Impact Assessment and convene a multi-stakeholder task team to define binding, NQF-aligned scopes of practice. This intervention is the decisive mechanism required to safeguard patient safety, validate professional integrity, and realize economic justice, ultimately elevating Somatology from a vulnerable trade into a respected, equitable, and healthcare-aligned profession.
